# The effect of perceived stress on depression in college students: The role of emotion regulation and positive psychological capital

**DOI:** 10.3389/fpsyg.2023.1110798

**Published:** 2023-03-13

**Authors:** Yafei Liu, Haibo Yu, Yaohui Shi, Chao Ma

**Affiliations:** ^1^Normal College, Shihezi University, Shihezi, China; ^2^Center of Application of Psychological Research, Shihezi University, Shihezi, China

**Keywords:** perceived stress, depression, emotion regulation, positive psychology capital, moderating effect

## Abstract

**Introduction:**

College students have become a high prevalence group and vulnerable group of depression. The present study aims to explore the effect of perceived stress on depression in a sample of Chinese college students and proposes that both emotion regulation and positive psychological capital play a moderating role between the two, so as to provide rational intervention for the prevention of potential depression among college students.

**Method:**

In this study, 1,267 college students (46.4% female) from a university in western China were selected for the study using a whole-group convenience sampling method.

**Results:**

After controlling for gender, this study found that both cognitive reappraisal and positive psychological capital positively moderated the relationship between perceived stress and depression, and both significantly inhibited depression in high and low stress perceivers, and the inhibitory effect was more pronounced in high stress perceivers, but expression inhibition did not moderate the relationship between perceived stress and depression.

**Discussion:**

The results suggest that college students can be helped to cope with the negative effects of perceived stress on depression by increasing the frequency of their use of cognitive reappraisal strategies and encouraging the accumulation of positive psychological capital. This study provides theoretical and practical implications for rational interventions for depression among college students.

## Introduction

Depression is a widespread mood disorder accompanied by key features such as depressed mood, reduced volitional activity, and reduced verbal actions (Smith and De Torres, 2014). It not only leads to impairment of social functions such as socialization and learning, but even triggers self-injurious and suicidal behaviors in individuals (Li et al., 2015). Depression is extremely common among the college student population (Liu et al., 2019). A Meta-analysis found a relatively high prevalence of depression among college students (31.38%) compared to the general population (5–6%) (Wang et al., 2020; Zhang et al., 2020). Recent studies have found that the prevalence of depression among Chinese college students reached 37.0% during the Covid-19 epidemic (Zhou et al., 2021). Notably, several studies have shown that depression causes severe impairments in cognitive and social functioning in some college students currently, such as decreased executive and memory functions, difficulty concentrating, and social avoidance (Rock et al., 2014), and individuals with severe depression are prone to severe insomnia, self-injury, and suicide due to their extreme psychological distress (Riemann et al., 2020). It is evident that current college students have become a high prevalence group of mental illness and a vulnerable group of mental health. Depression among college students deserves to be focused on because of its larger scope, deeper impact, and more serious outcome (Yang and Chen, 2015). Therefore, this study attempts to investigate the intrinsic regulatory mechanisms affecting depression among college students and provide intervention for the reasonable prevention of potential depression among college students.

## Literature review

### Perceived stress and depression

Perceived Stress is a stress perception that refers to an individual’s perception of the degree of stress caused by an external event, as well as the individual’s interpretation of the stressful event and perception of its objective existence as a variety of physical and mental tensions and discomforts (Cohen et al., 1983; Yang and Huang, 2003). Stress is seen as an important risk factor for mental health, and high stress perceptions often lead to a range of mental health impairments (O'Connor et al., 2021). The non-homeostatic model of stress suggests that the more stressful events an individual experiences and the longer they last, the more likely they are to perceive greater stress and the more likely they are to experience non-homeostatic states of mind and body, such as excessive emotional arousal (Yan et al., 2010). Empirical studies have shown that the higher the level of stress perception, the more likely it is to cause depression (Anyan and Hjemdal, 2016; Yang et al., 2020). Stressful events induce individuals’ perceived stress, especially in individuals who are more sensitive to stress. Perceptions of stress contribute to an increase in negative emotional experiences and a decrease in positive emotional experiences (Li et al., 2021) and a decrease in life satisfaction and psychological well-being (Fu et al., 2012), which in turn leads to depression (Li et al., 2015). In addition, compared to men, women are more sensitive to stress perception, who are less resilient, have higher emotional reactions to stress, have a more difficult time recovering from negative states, and are therefore at higher risk for depression (Liu et al., 2021). Therefore, it is necessary to explore the underlying psychological mechanisms between perceived stress and depression and then provide a scientific basis for the reasonable prevention of depression among college students.

### Moderating role of emotion regulation

Emotion regulation refers to the efforts of individuals to keep their emotions in a balanced and stable state to adapt and meet the needs of the social environment in a certain situation (Gross, 2015a). It is one of the important predictors of mental health by providing individuals with information related to their environment and protecting them from maintaining emotional balance (Khodami and Sheibani, 2020). The Emotion Regulation Process Model proposes that cognitive reappraisal and expression inhibition are two regulatory strategies that act on different emotion processing processes (Gross, 2015b). Several studies have found that greater use of cognitive reappraisal strategies implies a better state of mental health (Yang et al., 2020) and frequent use of expression-inhibitory strategies leads to increased psychological symptoms and impaired social functioning (Aldao et al., 2010; Lee et al., 2020). The interaction model of psychological resilience (Masten, 2001; Masten and Red, 2002) suggests that risk factors affect individuals less when protective factors are present than when they are not, that protective factors confer immunity to stress/adversity (the degree of immunity to individuals may vary across protective factors), and that protective factors influence the role of stress/adversity factors to psychosocial development through regulatory mechanisms. Research suggests that on a social reality level, individuals who use cognitive reappraisal strategies are more likely to share emotional experiences with others, build intimate relationships, and have more social support (Lu et al., 2019). Based on this, it is hypothesized that cognitive reappraisal acts as a buffer between perceived stress and depression in college students, and the interaction between protective factors (cognitive reappraisal) and risk factors (perceived stress) will reduce the possibility of adverse outcomes. When the frequency of cognitive reappraisal was low, the depression level of college students tended to increase significantly with the increase of perceived stress; when the frequency of cognitive reappraisal was high, the depression level of college students tended to increase slowly with the increase of perceived stress. In addition, cognitive reappraisal is a strategy to reduce negative emotional experiences with positive emotional connotations, whereas expression inhibition suppresses negative emotions, which remain (Lu et al., 2019). According to Li (2012) “provide timely help” model, individual resource factors moderate the relationship between risk factors and social adaptation, buffer or weaken the adverse effects of risk factors, and the development disadvantage of those with high risk compared to those with low ecological risk is reflected more in the case of low individual resources than in the case of high individual resources. Expression inhibition as a maladaptive emotion regulation strategy has been shown to consume more cognitive resources than adaptive emotion regulation strategies, as evidenced by inducing stronger peripheral physiological responses and limbic system activation (Yuan et al., 2014). Based on this, the present study hypothesized that the depression level of college students showed a significant upward trend with the increase of perceived stress when the frequency of expression inhibition use was high, and the depression level of college students decreased at a slower rate with the increase of perceived stress when the frequency of expression suppression use was low.

### Moderating role of positive psychological capital

Positive psychology reawakens focus on the positive qualities and good living of human being. In contrast to traditional cognitive theories of depression that focus on negative factors, positive psychology suggests that depression is caused by a lack of positive resources (Zhou et al., 2010). Positive psychological capital refers to a positive psychological state during an individual’s growth and development, including four core components of resiliency, optimism, hope, and self-efficacy (Ye and Fang, 2015), and effectively promote healthy behaviors by helping individuals cope with challenging environments (Cho et al., 2021). Also according to the interaction model of psychological resilience and the “provide timely help” model, psychological capital is a protective factor against depression in adolescents, and individuals with higher levels of psychological capital are able to use their positive abilities to cope with negative emotions, enhance their own protectiveness and adaptability to the outside world, and are less likely to experience emotional problems in risky stressful situations (Li et al., 2014). Individuals with lower levels of psychological capital, on the other hand, are more susceptible to their own state and may not be able to adequately mobilize internal resources to cope with negative emotions when faced with more stress, and thus may be more prone to emotional problems in risky stressful situations (Jeong and Jung, 2017; Turliuc and Candel, 2022). Based on this, it is hypothesized that positive psychological capital has a buffering effect on depression among college students. When the level of positive psychological capital is high, the depression level of college students tends to decrease significantly with the increase of perceived stress; when the level of psychological capital is low, the depression level of college students decreases slowly with the increase of perceived stress.

### The current study

In order to understand the effects of emotion regulation and positive psychological capital on the relationship between perceived stress and depression, a group of college students was selected as subjects in this study. Based on the “non-stationary” model of stress, the interaction model of psychological resilience, and the “provide timely help” model, we investigated the effects of perceived stress on depression, and further explored the moderating effects of emotion regulation and positive psychological capital on the relationship between perceived stress and depression in college students.

This study hypothesized that cognitive reappraisal could moderate the relationship between perceived stress and depression among college students, and that high-frequency cognitive reappraisal reduced the association between stress perception and depression, while low-frequency cognitive reappraisal enhanced the association between stress perception and depression (Hypothesis 1); Expression inhibition moderates the relationship between perceived stress and depression in college students, with high frequency of expression inhibition enhancing the association between stress perception and depression, and low frequency of expression inhibition decreasing the association between stress perception and depression (Hypothesis 2); positive psychological capital moderates the relationship between stress perception and depression in college students, with high level of positive psychological capital decreasing the association between stress perception and depression, and low level of positive psychological capital enhancing the association between stress perception and depression (Hypothesis 3).

## Materials and methods

### Participants

Using the whole group convenience sampling method, 1,312 questionnaires were distributed to all college students from freshman to senior students in a university in western China. After the questionnaires were returned, 45 invalid questionnaires were eliminated according to the criteria of invalid questionnaires if there were omissions or the same choice in multiple questions, and 1,267 valid questionnaires were obtained, with a valid return rate of 95.6%. Among them, 679 (53.6%) were male and 588 (46.4%) were female; 560 (44.2%) lived in rural areas and 707 (55.8%) lived in urban areas; 434 (34.3%) were only children and 833 (65.7%) were children with siblings; fathers’ education level: 208 (16.4%) were elementary school students and below, 540 (42.6%) were junior high school students, 297 (23.4%) were senior high school (or technical school) students, 214 (16.9%) were undergraduates (or junior college students) and 8 (0.6%) were graduate students and above; mothers’ education level: 352 (27.8%) were elementary school students and below, 468 (36.9%) were junior high school students, 279 (22.0%) were senior high school (or technical school) students, 162 (12.8%) were undergraduates (or junior college students), and 6 (0.5%) were graduate students and above. The study was approved by the Science and Technology Ethics Committee of the First Affiliated Hospital of Shihezi University School of Medicine (approval number: KJ2022-152-01), and all investigated college students gave their informed consent.

### Measurements

#### Perceived stress scale

The Chinese version of Perceived Stress Scale developed by Cohen et al. and revised by Yang and Huang (2003) was used, consisting of 14 items, including two facets, tension (e.g., “feeling tense and stressed”) and loss of control (feeling unable to control the important things in one’s life), which are used to detect the overall and prevalent stress in life. It indicates a degree of self-awareness. Participants rated each item on a 5-point likert scale ranging from 1 (*strongly disagree*) to 5 (*strongly agree*), with higher scores indicating greater perceived psychological stress. The scale has good reliability and validity, and the Cronbach’s alpha coefficient in this study was 0.71.

#### Emotion regulation questionnaire

The Chinese version of the Emotion Regulation Questionnaire, developed by Gross and revised by Wang et al. (2007), was used to assess the frequency of emotion regulation among college students. It includes 10 items, divided into two dimensions: cognitive reappraisal and expression inhibition. Cognitive reappraisal is a strategy for shifting situationally evoked emotions before they arise (e.g., “I control my emotions by changing the way I think about the situation”), and expression inhibition is a strategy for individuals to inhibit the expression of emotions after they arise (e.g., “I control my emotions by not expressing them”) (Ogbaselase et al., 2022). The 7-point likert scale was used ranging from 1 (*strongly disagree*) to 7 (*strongly agree*), with higher scores on the dimension indicating that the individual uses the strategy more frequently. The reliability of the scale was good. The Cronbach’s alpha coefficients for cognitive reappraisal and expression suppression in this study were 0.85 and 0.60, respectively, and the Cronbach’s coefficient for the total scale was 0.80.

#### Positive psychological capital questionnaire

The Positive Psychological Capital Questionnaire developed by Zhang G et al. (2010) was used to assess the level of positive psychological capital development among college students (e.g., “When the situation is uncertain, I always expect a good outcome”). It includes 26 items divided into four facets: self-efficacy, resilience, hope, and optimism. The 7-point likert scale was used from 1 (*not at all likely*) to 7 (*highly likely*), and the higher the scores, the better the psychological capital development of the individual. The reliability of the questionnaire was good, and the Cronbach’s alpha coefficient was 0.93 in this study.

#### The Center for Epidemiological Studies Depression Scale

The Center for Epidemiological Studies Depression Scale developed by Radloff and revised by Zhang J et al. (2010), was used to examine individuals’ depressed mood status (e.g., “I feel that I cannot get rid of this misery even with the help of my lover or friends”) over a 1-week period. It includes 20 items divided into four facets: depressed mood, somatic symptoms, positive mood, and interpersonal relationship. A 4-point likert scale was used from 0 (*occasionally or not, less than one day*) to 3 (*most of the time, 5–7 days/week*), with higher scores indicating more severe depression. The reliability of the scale was good, and the Cronbach’s alpha coefficient in this study was 0.88.

#### Procedure

College students at a university in western China were used as the subjects and the investigators were well-trained graduate students. Informed consent was signed prior to administration, a group instruction was read out explaining the purpose of the study and promising to keep the subjects’ responses confidential, and independent responses and silence were emphasized during the administration of the test. The administration process took approximately 20 min, and all questionnaires were collected on site.

#### Data analysis

Data were analyzed using SPSS 21.0 software, and Pearson product difference correlation analysis was used to predict the correlation of key variables: perceived stress, emotion regulation, positive psychological capital, and depression. RStudio software (R version 3.3.1) was used to apply the process and combine the selected point method and Johnson-Neyman method to test for moderating and simple effects.

## Results

### Common method bias test

In this study, common method bias was controlled by measures such as anonymous measurement and partial item reversal (Zhou and Long, 2004), but the problem of common method bias may still exist, so the Harman’s one-way method was used to test for common method bias. Exploratory factor analysis of perceived stress, cognitive reappraisal, expression inhibition，positive psychological capital, and depression showed that unrotated factor analysis indicated 12 common factors with eigenvalues greater than 1. The first factor explained only 26.80% of the overall variance, well below the threshold of 40%, indicating that the study did not have common method bias problems of a more serious nature.

### Descriptives and correlations

To understand gender differences in perceived stress, cognitive reappraisal, expressive inhibition, positive psychological capital, and depression among college students, independent samples t-tests were conducted to examine perceived stress, cognitive reappraisal, expressive inhibition, positive psychological capital, and depression among college students. The results showed that there were significant differences in perceived stress between male and female students (t = −2.42, *p* < 0.05, Cohen’s d = −0.15), and male students’ perceived stress (M = 2.68, SD = 0.46) was significantly lower than that of female students (M = 2.74, SD = 0.44); male and female students differed significantly in expression inhibition (t = 6.91, *p* < 0.001, Cohen’s d = 0.39), with male students expressing significantly more inhibition (M = 4.60, SD = 0.95) than female students (M = 4.24, SD = 0.90); male and female students differed significantly in positive psychological capital (t = 2.52, p < 0.05, Cohen’s d = 0.14), with male students having significantly higher positive psychological capital (M = 4.99, SD = 0.82) than female students (M = 4.88, SD = 0.76); however, the differences in cognitive reappraisal and depression between male and female students were not significant (*p* > 0.05).

The results of descriptive statistics and correlation matrix analysis for each variable are shown in Table 1. perceived stress, cognitive reappraisal, expression inhibition, positive psychological capital, and depression were significantly correlated with each other, except for expression inhibition, which was not correlated with perceived stress and depression. In addition, gender was significantly and positively correlated with perceived stress, and negatively correlated with expression inhibition and positive psychological capital, while other additional variables (including family residence and parental education level) were not correlated with the predictor variables. Based on this, gender was used as a control variable in the subsequent test analysis.

**Table 1:** Descriptive analysis and correlation coefficients of the study variables.

variables	*M ± SD*	1	2	3	4	5	6
1. Gender		1					
2. Perceived stress	2.71 ± 0.45	0.07*	1				
3. Cognitive appraisal	4.98 ± 0.87	−0.03	−0.31^**^	1			
4. Expression inhibition	4.43 ± 0.94	−0.19**	−0.03	0.39^**^	1		
5. Positive psychological capital	4.94 ± 0.80	−0.07*	−0.61^**^	0.59^**^	0.15**	1	
6. Depression	1.51 ± 0.40	−0.04	0.59**	−0.36^**^	−0.01	−0.63**	1

Gender was a dummy variable coded as 1 = male and 2 = female.

*The correlation is significant at the 0.05 level.

**The correlation is significant at the 0.01 level.

### Analysis of the moderating effects of emotion regulation and positive psychological capital

Prior to further analysis, all predictor variables were standardized. Given that the moderating variables were all continuous, this study used RStudio to apply process (Model 1) to analyze the standardized moderating effects. Results showed that the model for the moderating effect of cognitive reappraisal between perceived stress and depression was significant *F*(4,1,262) = 220.40, *p* < 0.001, with a model explained R^2^ of 0.41. After controlling for the additional variable gender, perceived stress significantly predicted depression positively (β = 0.53, *t* = 23.44, *p* < 0.001), with a confidence interval of [0.49, 0.58]. Cognitive reappraisal significantly negatively predicted depression (β = −0.21, *t* = −9.23, *p* < 0.01) with a confidence interval of [−0.25, −0.17]; and the interaction term for perceived stress and cognitive reappraisal was significant (β = −0.12, *t* = −6.31, *p* < 0.001), with a confidence interval of [−0.15, −0.08], indicating a significant moderating effect of cognitive reappraisal between perceived stress and depression; however, the moderating effect of expression inhibition between perceived stress and depression was significant *F*(4,1,262) = 176.23, *p* < 0.001, with a model explained *R*^2^ of 0.36. After controlling for the additional variable gender, perceived stress was a significant positive predictor of depression (β = 0.60, *t* = 26.32, *p* < 0.001) with a confidence interval of [0.55, 0.64]; expression inhibition failed to predict depression significantly (β = −0.01, *t* = −0.47, *p* > 0.05) with a confidence interval of [−0.06, 0.03]; and the interaction term between perceived stress and expression inhibition was also not significant (β = −0.04, *t* = −1.93, *p* > 0.05) with a confidence interval of [−0.08, 0.00], indicating that the moderating effect of expression inhibition between perceived stress and depression was not significant. In addition, the model for the moderating effect of positive psychological capital between perceived stress and depression was significant, *F*(4,1,262) = 315.91, *p* < 0.001, with a model explanatory R^2^ of 0.50. After controlling for the additional variable gender, perceived stress significantly positively predicted depression (β = 0.34, *t* = 13.51, *p* < 0.001), with a confidence interval of [0.29, 0.39], positive psychological capital significantly negatively predicted depression (β = −0.43, *t* = −17.11, *p* < 0.001), with a confidence interval of [−0.48, −0.38], and the interaction term between perceived stress and positive psychological capital was significant (β = −0.13, *t* = −8.21, *p* < 0.001), with a confidence interval of [−0.16, −0.10], indicating a significant moderating effect of positive psychological capital between perceived stress and depression.

**Figure 1:**
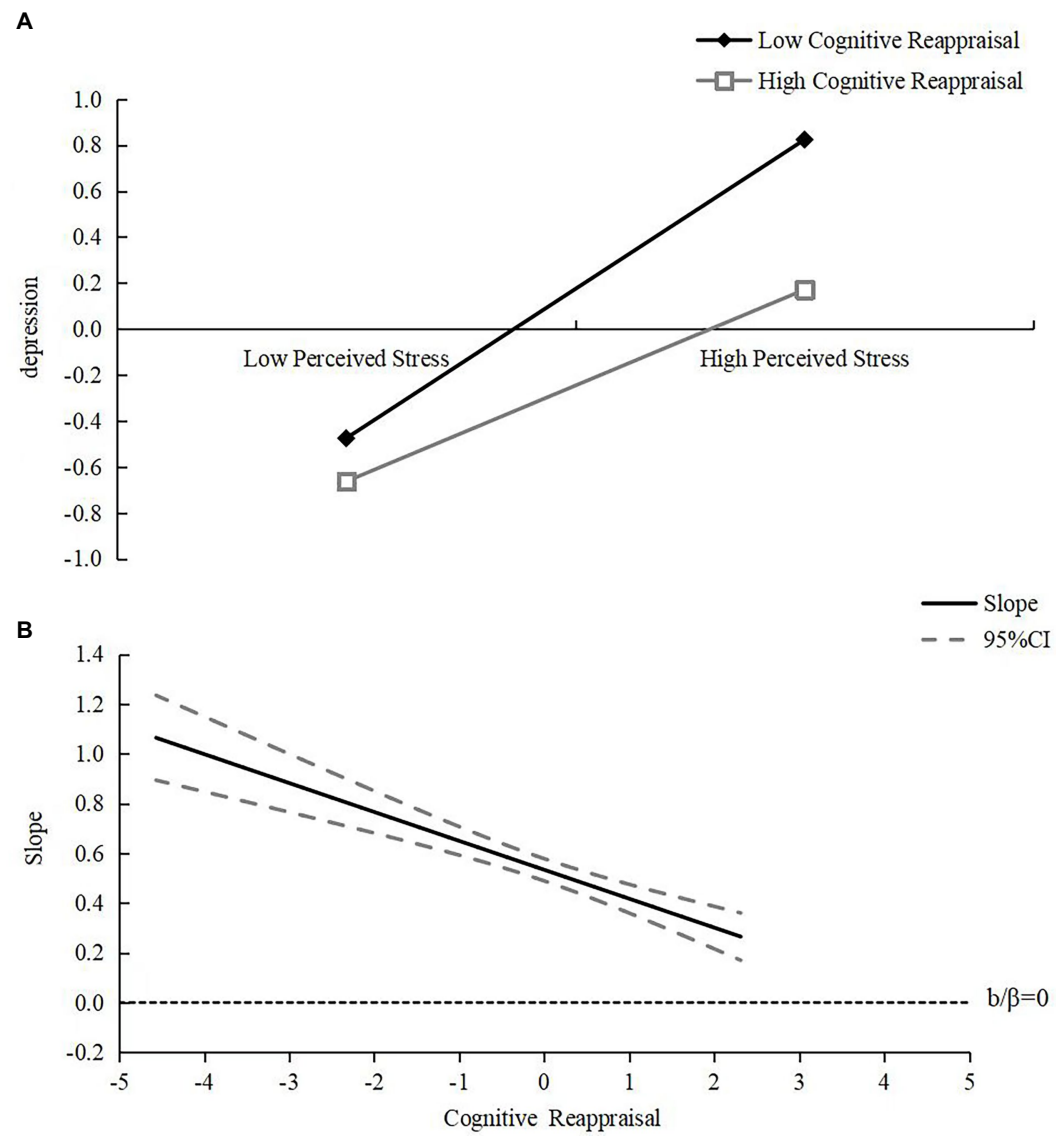
The moderating role of cognitive reappraisal in the relationship between perceived stress depression **(A)** and change of its simple slope **(B)**.

The selected point method and Johnson-Neyman method are commonly used to conduct simple slope tests. The Johnson-Neyman method can overcome the limitation that the selected-point method can only test one value of the moderating variable at a time by fixing the t-value as the critical value and finding the range of values of the moderating variable when the simple slope is significantly non-zero (Fang et al., 2015). In order to clarify the moderating effects of cognitive reappraisal and positive psychological capital on perceived stress and depression among college students, this study used both methods to conduct simple slope tests simultaneously. For cognitive reappraisal, the results of the selected-point method analysis revealed that cognitive reappraisal significantly and positively predicted depression in both the high stress perception group (M + 1SD, i.e., one standard deviation above the mean) and the low stress perception group (M-1SD, i.e., one standard deviation below the mean) (*β_high perceived stress group_* = 0.42, *t_high perceived stress group_* = 14.20, *p < 0*.001; *β_low perceived stress group_* = 0.65, *t_low perceived stress group_* = 22.28, p < 0.001) (see Figure 1A). At the same time, the Johnson-Neyman method analysis revealed that the simple slope was significantly non-zero over the range of values of cognitive reappraisal [−4.56, 2.31] (after standardization) (see Figure 1B). The higher the level of perceived stress, the stronger the effect of cognitive reappraisal in negatively predicting depression, i.e., the value of the inhibitory effect of cognitive reappraisal on depression gradually increases as perceived stress rises.

**Figure 2:**
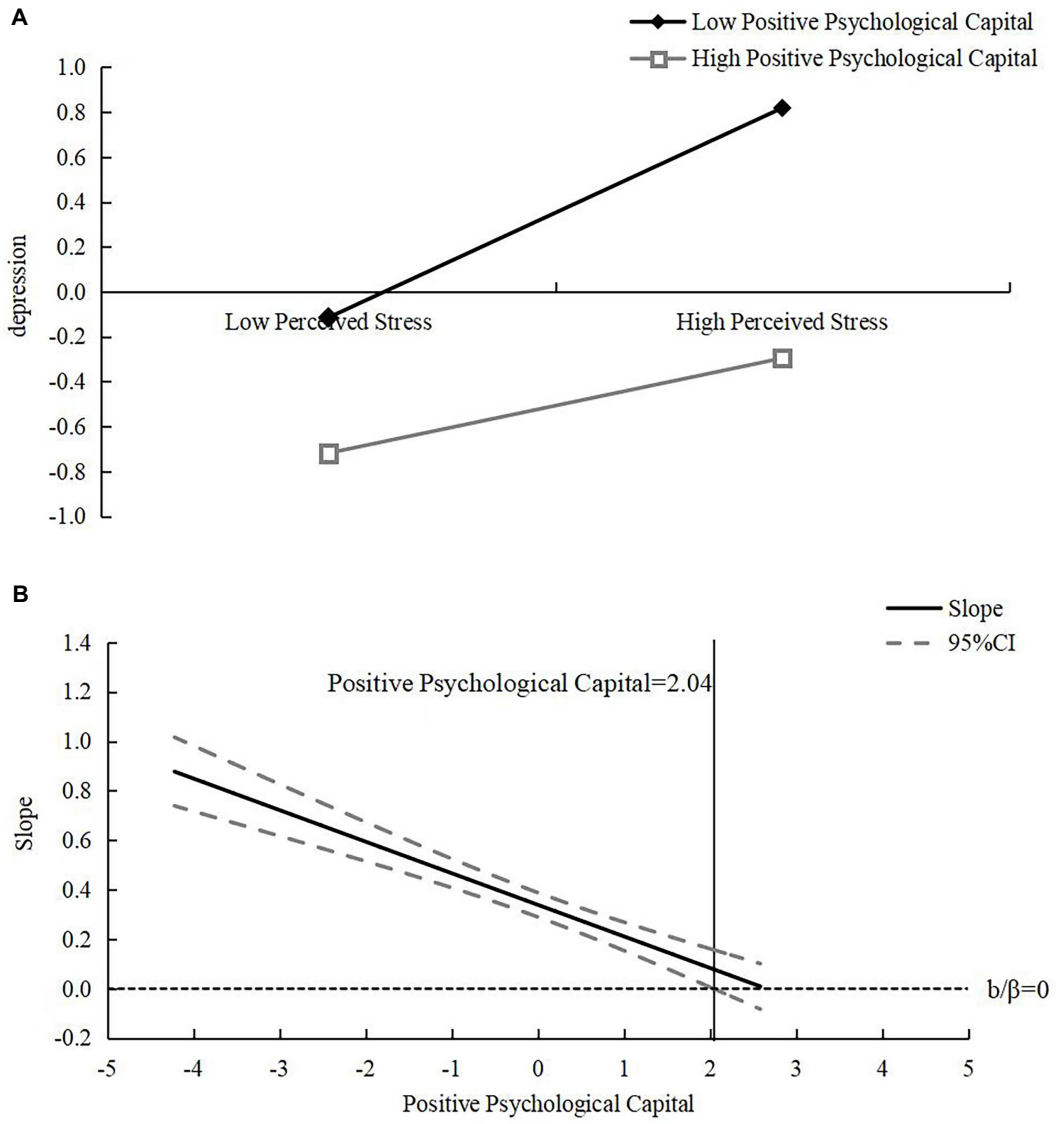
The moderating role of positive psychological capital in the relationship between perceived stress and depression **(A)** and change of its simple slope **(B)**.

For the moderating effect of positive psychological capital, the results of the selected-point method analysis found that positive psychological capital significantly and positively predicted depression in both the high stress perception group (M + 1SD) and the low stress perception group (M-1SD) (*β_high perceived stress group_* = 0.21, *t_high perceived stress group_* = 7.23, *p <* 0.001; *β_low perceived stress group_* = 0.47, *t_low perceived stress group_* = 15.67, *p <* 0.001) (see Figure 2A). Meanwhile, the Johnson-Neyman method analysis found that the confidence interval of the simple slope did not contain 0 within the range of values of positive psychological capital [−4.21, 2.04] (after standardization) (see Figure 2B), and the simple slope was significant. Also, in this range, the higher the level of perceived stress, the stronger the effect of positive psychological capital in negatively predicting depression, i.e., as perceived stress rises, the value of the inhibitory effect of positive psychological capital on depression gradually increases.

## Discussion

### The relationship between perceived stress and depression among college students

The present study found that gender did not differ in depression levels among college students, which is not consistent with the results of previous studies (Liu et al., 2021). Existing studies have generally concluded that there are gender differences in depression, and that the differences in depression by gender are reflected in many aspects such as influencing factors, onset symptoms, age of onset, and incidence of depression (Ren et al., 2019). However, a recent study found cross-gender consistency between individual genders in network structure, network connection strength, and core symptoms based on the network analysis level, possibly implying mutual independence between depression networks and total depression scores (Huang et al., 2022). This explains the possibility that gender does not differ in depression levels among college students, but follow-up studies are still needed to verify this.

Previous studies have shown that individuals who are more sensitive to perceived stress have higher levels of depression (Zandifar et al., 2020). The present study found that perceived stress positively influenced depression among college students, suggesting that perceived stress is an important predictor of depression among college students. Thus, the result of this study is consistent with previous studies. Study the reasons, on the one hand, physiological evidence of neurohumoral mechanisms suggests that psychological stress activates the thalamic-pituitary–adrenal axis, releasing major neurotransmitters of stress, such as adrenaline, while inducing a series of peripheral responses to negative emotions (i.e., anger and fear). In contrast, the emergency state prompts the release of central adrenaline, which stimulates the main neurotransmitter of depressed mood, pentraxin, and ultimately leads to the central production of depressed mood (Zheng et al., 2015). This also confirms the non-homeostatic model of stress. On the other hand, the quality-stress interaction model suggests that the properties of the event itself and psychological susceptibility are the determinants of the emergence of depression and anxiety after a negative life event, where psychological susceptibility emphasizes that the specific environment is more likely to activate the individual’s susceptibility. The psychological susceptibility emphasizes that a particular environment is more likely to activate the susceptible qualities of an individual (Rosenthal, 1963). According to this theory, the stronger the individual’s perception of stress, the more likely he or she is to perceive or notice threatening stimuli in the external environment, which causes psychological tension and discomfort and contributes to a recurrent cycle of negative emotions, leading to higher levels of depression (Yuan, 2016). Both evidence from neurohumoral mechanisms and theoretical interpretations of environmental formation suggest that perceived stress is a booster for predicting psychological problems such as depression in individuals (Yang et al., 2020). Therefore, it is important to minimize the impact of stress on depression in college students and to improve their mental health.

### Moderating effects of emotion regulation and positive psychological capital between perceived stress and depression among college students

In this study, we found that both cognitive reappraisal and positive psychological capital positively moderated the relationship between perceived stress and depression among college students. The positive predictive effect of cognitive reappraisal and positive psychological capital on depression was stronger when the level of perceived stress was higher, i.e., the inhibitory effect of cognitive reappraisal and positive psychological capital on depression was more obvious as the level of perceived stress increased. This may be due to the fact that cognitive reappraisal and positive psychological capital, as a protective factor, can provide individuals with useful help and support to mitigate the effects of perceived stress on depression, enhance the ability to adapt to high-pressure environments, and resist the generation of negative emotions such as depression. This suggests that cultivating and improving cognitive reappraisal strategies and positive psychological capital can be more effective in reducing the risk of depression among high perceived stress college students and also help to reduce the likelihood of depression among low perceived stress college students.

Notably, the present study found that expressive inhibition failed to moderate the relationship between perceived stress and depression in college students. Previous studies have found that expression inhibition inhibits emotions at the behavioral level and does not promote sympathetic nervous system activity to mobilize the potential of multiple organs of the individual to adapt to environmental changes (Huang et al., 2021). It has been shown that cognitive reappraisal occurs early in the process of emotion generation and intervenes before the tendency to respond to emotions is fully developed, and is effective in changing subsequent emotional trajectories, compared to expression inhibition, which is effective in controlling and reducing the expression of emotional behaviors but not the frequency of emotional experiences (Gross and John, 2003). Thus individuals who exhibit depressive symptoms are more likely to overuse expression inhibition to cope with negative events, but good use of cognitive reappraisal can help them mitigate and shorten the cycles, thereby reducing vulnerability to depressive impairment (Balderas et al., 2021). The results of this study further illustrate the effectiveness and adaptability of cognitive reappraisal strategies in regulating stress perceptions and depression in college students.

In addition, this study found that cognitive reappraisal and positive psychological capital had similar effects, but the inhibitory effects of cognitive reappraisal and positive psychological capital on perceived stress and depression differed in two ways. On the one hand, the simple slope analysis of the selected point method shows that the slope of cognitive reappraisal is greater, more effective and more efficient than that of positive psychological capital in suppressing depression in college students. On the other hand, the simple slope of the Johnson-Neyman method shows that the effect of cognitive reappraisal on depression is better than that of positive psychological capital, and the effect can be achieved from −4.56 standard scores of perceived stress, indicating that college students with low perceived stress below zero standard scores can be protected by cognitive reappraisal. The importance of cognitive reappraisal for college students’ mental health is slightly better than that of positive psychological capital. This may be due to the fact that cognitive reappraisal can improve the effect of perceived stress on depression by changing the poor perception of the cause of emotional events and using positive thinking to explain the negative emotional events they face, which can lead to more positive emotional experiences and increase happiness and life satisfaction, and then further promote the improvement of positive psychological capital on this basis.

### Implications, limitations, and future directions

This study innovatively used both the selected point method and Johnson-Neyman method to test the simple slope, and more clearly verified the predictive role of perceived stress on college students’ depression and the buffering role of emotion regulation and positive psychological capital in between, which has some implications for colleges to pay attention to students’ mental health and develop intervention programs. First, since perceived stress can positively predict depression among college students, society and colleges should pay attention to reducing college students’ stress and improving their ability to cope with stress, so as to reduce their risk of depression. Secondly, cognitive reappraisal and positive psychological capital can effectively reduce the risk of depression in high perceived stress. Therefore, mental health education in colleges and universities should focus on cultivating and enhancing students’ ability to use adaptive emotion regulation strategies and encouraging and guiding the accumulation of positive psychological capital in order to effectively reduce the risk of depression in high perceived stress college students and prevent the possibility of depression in low perceived stress college students. Finally, cognitive reappraisal is more effective in inhibiting high perceived stress college students, so more attention should be paid to cultivating college students’ ability to use cognitive reappraisal strategies to help them have more positive emotions to resist stress risks in various stressful situations.

There are two shortcomings in this study: on the one hand, considering that the current sample was taken from a group of college students in a western university, the sampling range is slightly narrow, and the generalizability of the results needs to be tested. Future studies can be conducted on a nationwide basis with a large sample. On the other hand, the data in the current study are cross-sectional data, and it is not possible to determine the causality of each variable in a strict sense. Future studies could combine cross-lagged designs and experimental studies to investigate the causal mechanisms between the two.

## Conclusion

The present study found that both cognitive reappraisal and positive psychological capital positively moderated the relationship between perceived stress and depression, and both significantly inhibited depression in high and low stress perceivers, and the inhibitory effect was more pronounced in high stress perceivers, but expression inhibition could not moderate the relationship between perceived stress and depression.

## Data availability statement

The raw data supporting the conclusions of this article will be made available by the authors, without undue reservation.

## Ethics statement

The studies involving human participants were reviewed and approved by Science and Technology Ethics Committee of the First Affiliated Hospital of Shihezi University School of Medicine. The patients/participants provided their written informed consent to participate in this study.

## Author contributions

YL and HY designed the experiment, collected data, prepared the manuscript, and made data analysis. YS corrected the whole language of the manuscript and made final approval. CM gave technique supports and valuable suggestions in experiment designing. All authors contributed to the article and approved the submitted version.

## Funding

This research was supported by the Shihezi University Graduate Education Teaching Reform Research Project of China (2021Y-JGSJ11).

## Conflict of interest

The authors declare that the research was conducted in the absence of any commercial or financial relationships that could be construed as a potential conflict of interest.

## Publisher’s note

All claims expressed in this article are solely those of the authors and do not necessarily represent those of their affiliated organizations, or those of the publisher, the editors and the reviewers. Any product that may be evaluated in this article, or claim that may be made by its manufacturer, is not guaranteed or endorsed by the publisher.
